# INTERPROFESSIONAL ROLES AND COLLABORATIONS TO ADDRESS COVID-19 PANDEMIC CHALLENGES IN NURSING HOMES

**DOI:** 10.24926/ijps.v9i1.4644

**Published:** 2022-05-24

**Authors:** Yu Jin Kang, Karen A. Monsen, Betsy Jeppesen, Candy Hanson, Kathie Nichols, Kelly O’Neill, Jennifer Lundblad

**Affiliations:** Nell Hodgson Woodruff School of Nursing, Emory University; University of Minnesota School of Nursing; Stratis Health; Stratis Health; Stratis Health; Stratis Health; Stratis Health

**Keywords:** COVID-19 pandemic, Interprofessional and multidisciplinary partnership, Nursing home, The Omaha System

## Abstract

Nursing home experts and informatics nurses collaborated to develop guidelines for nursing homes that revealed partnership principles in action during the COVID-19 pandemic. This article describes efforts to define interprofessional nursing home staff roles within the partnership-based COVID-19 Response Guideline, and to examine changes in nursing practice compared to the pre-pandemic practice of nurses. The qualitative process of identification of nursing home staff roles revealed the extensive scope of interprofessional partnership needed to respond to the pandemic. Using the Omaha System structure, we compared these collective COVID-19 response interventions of Nursing Service roles with nursing interventions of RNs and LPN/LVNs defined in previous nursing home studies. This comparison showed the necessary transformation and collaboration among nurses needed for the pandemic response in nursing homes. The Omaha System Pandemic Guideline is available online and in the Omaha System Guidelines app for immediate use as COVID-19 response practice guidelines and references for interprofessional roles in nursing homes, as well as for multidisciplinary roles across diverse care settings. The guideline is an exemplar of how informatics can facilitate interprofessional and multidisciplinary partnership for nursing homes and other care settings. Future use of the guidelines for decision making and documentation related to infection prevention and control in nursing homes may improve care quality and health outcomes of residents and population.

## BACKGROUND

Early research demonstrated the vulnerability of the nursing home population to Coronavirus Disease 2019 (COVID-19), showing higher rates and more rapid spread of COVID-19 cases than in the general population (Yi et al., 2020; Grabowski & Mor, 2020). In addition to insufficient financial and human resources and supplies, congregate living and physical layouts made nursing homes more vulnerable to COVID-19, turning them into ground zero for the COVID-19 pandemic (Bakerjian et al., 2021; Barnett & Grabowski, 2020; Davidson & Szanton, 2020; Grabowski & Mor, 2020; Kolanowski et al., 2021). A new care model was needed immediately to support nursing home employees, who were struggling to achieve infection control and manage the crisis with limited resources (Grabowski & Mor, 2020; Kolanowski et al., 2021; Levine et al., 2020; Stall et al., 2020; White et al., 2021).

To help mitigate this abrupt and radical shift, nursing home experts and informatics nurses adopted the perspective of partnership systems, which pursues societies based on mutual respect, responsibility, and caring relationships (Eisler, 1989). In contrast to top-down domination systems, partnership systems encourage mutual respect, responsibility, and benefits across hierarchies (Eisler, 2021). Nursing homes are a highly regulated environment with hierarchical care roles delineated by federal and state laws (Requirements for States and Long Term Care Facilities, 2015). Prior to the pandemic, each discipline had separate care goals and carried out different and clearly specified responsibilities for resident care. However, as the pandemic unfolded, nursing home staff combined forces to integrate care responsibilities across roles to save lives and deliver quality resident care.

### The Omaha System Guidelines

When COVID-19 was declared a pandemic by the World Health Organization (WHO) in March 2020, the Omaha System-encoded evidence-based multidisciplinary guideline for the COVID-19 response was developed based on U.S. Centers for Disease Control and Prevention (CDC) and WHO sources (Monsen, 2020). This COVID-19 Response Guideline was an extension project of the Omaha System Guidelines project, which had been initiated to encode and disseminate evidence-based multidisciplinary practices for client care (Monsen et al., 2011; Monsen et al., 2012; Omaha System Guidelines, 2021; Slipka & Monsen, 2018; Monsen, 2020). Across health care settings, the COVID-19 pandemic impacted care environments and forced staff to adapt under limited resources, with uncertain information. The COVID-19 Response Guideline sought to address this issue by establishing interprofessional partnerships and providing recommendations for evidence-based infection prevention and control practice. Building on this body of work, nursing home experts and Omaha System researchers collaborated to define the COVID-19 Response Guideline for resident care in nursing homes. The Omaha System Guidelines, which include the COVID-19 Response Guideline, are publicly available in both web and app versions for iPhone and Android, on iTunes and Google Play (Omaha System Guidelines, 2021).

### The Omaha System and its Applications in Nursing Home Practice

**Figure 1:**
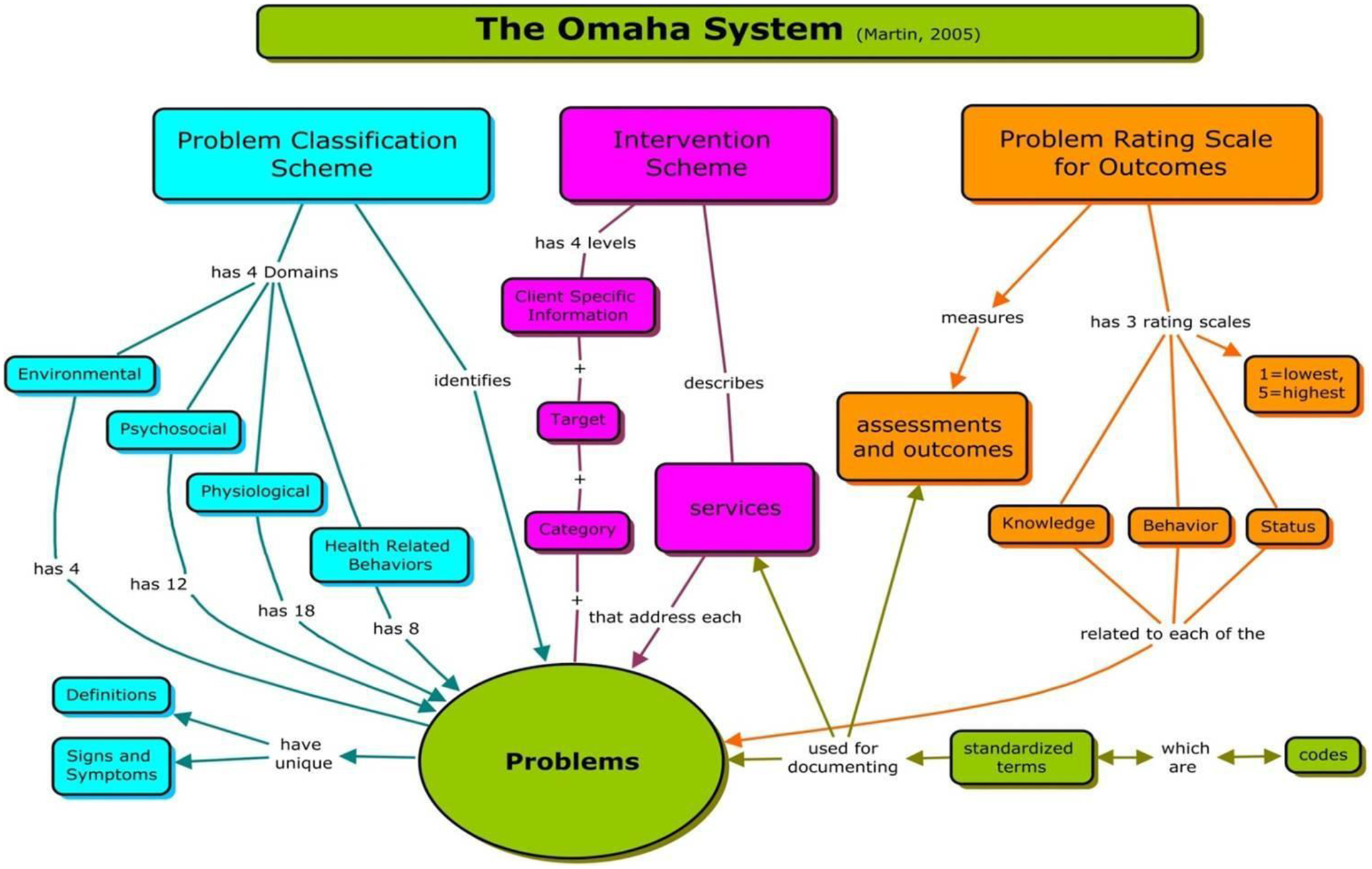
Concept Map of The Omaha System Note; Copyright Karen A. Monsen, 2009, used with permission

The Omaha System is a standardized terminology for comprehensive practice, documentation, and information management of client care that has been recognized by the American Nurses Association since 1992 (Martin, 2005). It consists of three related components: a Problems Classification Scheme, an Intervention Scheme, and a Problem Rating Scale for Outcomes, all of which enable a comprehensive health assessment and description of multidisciplinary practices across healthcare settings (Martin, 2005) (see Figure 1). It is available in the public domain and has been widely used in community care settings, generating valuable data through documentation during the course of routine practice (Monsen et al., 2019).

Prior to the COVID-19 pandemic, a study using the Omaha System defined 57 nursing interventions observable in registered nurses (RNs) and licensed practical nurses/licensed vocational nurses (LPN/LVNs) practicing in nursing homes (Kang et al., in press, see Appendix 1). These interventions were used as the basis of a time and motion study to observe workflow in a nursing home (Kang et al., 2021). The study showed that RNs and LPN/LVNs focused mainly on medications, communication with the care team, and conversations with residents and family, but relatively less on infection control and prevention (Kang et al., 2021). The study also confirmed a high degree of time pressure with a median intervention time of 32 seconds, an average of 66 interventions and 28 location changes per hour, and multitasking for 30% of total intervention time (Kang et al., 2021).

At the inception of the COVID-19 outbreak, significant changes in interventions for resident care would be expected, to implement infection prevention and control training and measures (Scopetti et al., 2021). In addition to the increasing work demands, nurse shortages and lack of RNs with geriatric nursing and leadership competencies further exacerbated nursing workload (Bakerjian at al., 2021; Davidson & Szanton, 2020; Kolanowski et al., 2021; Scopetti et al., 2021; White et al., 2021). Therefore, fostering cultural change from patterns of domination to relationships of partnership among nursing home staff was critical for successful management and surveillance of COVID-19 (Kennedy Oehlert, 2015).

Nursing home experts and Omaha System researchers collaborated to support this cultural change. They identified and defined the interprofessional nursing home staff roles for the collective COVID-19 response. They also investigated changes in the care responsibilities of RNs and LPN/LVNs during the COVID-19 pandemic compared to responsibilities defined prior to the pandemic. This article describes efforts to define interprofessional nursing home staff roles within the partnership-based COVID-19 Response Guideline, and to examine changes in nursing practice from pre-pandemic practice of RNs and LPN/LVNs.

## METHODS

### Aim 1: Define Interprofessional Nursing Home Staff Roles

The multidisciplinary COVID-19 Response Guideline was developed using a crowdsourcing technique over a series of webinars with diverse content experts across healthcare roles and settings (Monsen et al., 2021). Crowdsourcing refers to a collective, voluntary online activity opened by various entities calling for contributions from individuals with varying knowledge, experiences, and resources for mutual benefit (Estellés-Arolas & González-Ladrón-De-Guevara, 2012). The content experts reviewed the most recent evidence-based guidance from CDC and WHO sources (CDC, 2021; WHO, 2021); abstracted relevant content; mapped the content to Omaha System terms; and validated the mapped content from the team and public comments during the Omaha System Community of Practice international webinars attended by more than 400 individuals from 22 countries. For this study, nursing home experts (CH, KN, KO) and experts in informatics and the Omaha System (YK, KAM) used crowdsourcing techniques to identify a partnership-based interprofessional response to COVID-19 in nursing homes and to achieve consensus on it before incorporating the interventions into the COVID-19 Response Guideline.

First, the nursing home experts reviewed the content from the CDC and WHO resources mapped to the existing multidisciplinary roles within the COVID-19 Response Guideline. The nursing home experts from the Stratis team had deep expertise and extensive work experience in nursing homes, and provided technical assistance with infection prevention and control beginning with the onset of the pandemic. To develop interprofessional nursing home staff roles for the COVID-19 response, the Stratis team identified specific needs in the nursing home setting for care delivery and efficient workflow. The Stratis team approached multiple dimensions of staff roles: existing nursing-specific roles, professionals and paraprofessionals, and various job titles, and identified six key roles involved in partnership: Administration, Nursing Services, Therapeutic Services, Dietary Services, Contract Services and Environmental Services (see Table 1).

Next, the Stratis team and the Omaha System researchers employed a two-phase qualitative process (mapping and expert consensus) to define collaborative nursing home staff practice for the COVID-19 Response Guideline. Each nursing home expert independently mapped all activities in the guideline to the scope of practice for each of the proposed nursing home staff roles, assuming they were practicing to the top of their license or certification. All experts then shared and discussed their independent findings until consensus about the roles and interventions was achieved. The results were proposed to the Omaha System Community of Practice during an international webinar, consistent with the crowdsourcing techniques used to develop the COVID-19 Response Guideline.

### Aim 2: Examine Changes in Nursing Practice Compared to the Pre-pandemic Practice of RNs and LPN/LVNs

To examine the changes in nursing practice during the COVID-19 pandemic, the Nursing Services role defined in the COVID-19 Response Guideline by the Stratis team was compared to the pre-pandemic nursing practice defined in the previous study (Kang et al., in press). The Nursing Services role was based on partnerships among RNs, LPNs/LVNs, and certified nursing assistants (CNAs). Patterns of domination existed among them, but the guideline attempted to empower them within their legal scope of practice and emphasize their collaborative efforts.

#### Instrument.

Pre-pandemic practice of RNs and LPN/LVNs in nursing homes, referred to as the Omaha System interventions, was encoded using the Problem Classification Scheme and the Intervention Scheme of the Omaha System (Kang et al., in press, Appendix 1). The Problem Classification Scheme includes 42 health-related concepts in four domains (Environmental, Psychosocial, Physiological, and Health-related Behaviors) (Martin, 2005). The Intervention Scheme describes interventions in a multi-level hierarchy of categories (priority areas of practice), targets (care action), and care descriptions (further specifying the action) (Martin, 2005; Monsen et al., 2011). Each evidence-based intervention is encoded with a single problem, category, target term, and specific care description for resident care.

## RESULTS

### Aim 1: Define Interprofessional Nursing Home Staff Roles

A new set of six nursing home staff roles and 112 interventions encoded using the Omaha System was added to the COVID-19 Response Guideline (see Table 2). Of a total of 117 interventions included in the guideline (Monsen et al., 2021), 96% were within scope for at least one of the nursing home roles. Interventions varied by role, with 38% deemed within scope for all six roles. The complete COVID-19 Response Guideline is available on the Omaha System Guidelines website and app (Omaha System Guidelines, 2021; Monsen et al., 2021).

The nursing home COVID-19 response interventions addressed all Omaha System categories (care areas): *Teaching, Guidance, and Counseling* (30%), *Case Management* (28%), *Treatments and Procedures* (26%), and *Surveillance* (16%). The interventions addressed 29 of the 75 Omaha System targets (care actions); most frequent were *infection precautions* (24%), *medication coordination* (9), *sickness/injury care* (7%), *medical/dental care* (4%), *interaction* (4%), and *coping skills* (4%).

### Aim 2: Examine Changes in Nursing Practice Compared to the Pre-pandemic Practice of RNs and LPN/LVNs

**Figure 2:**
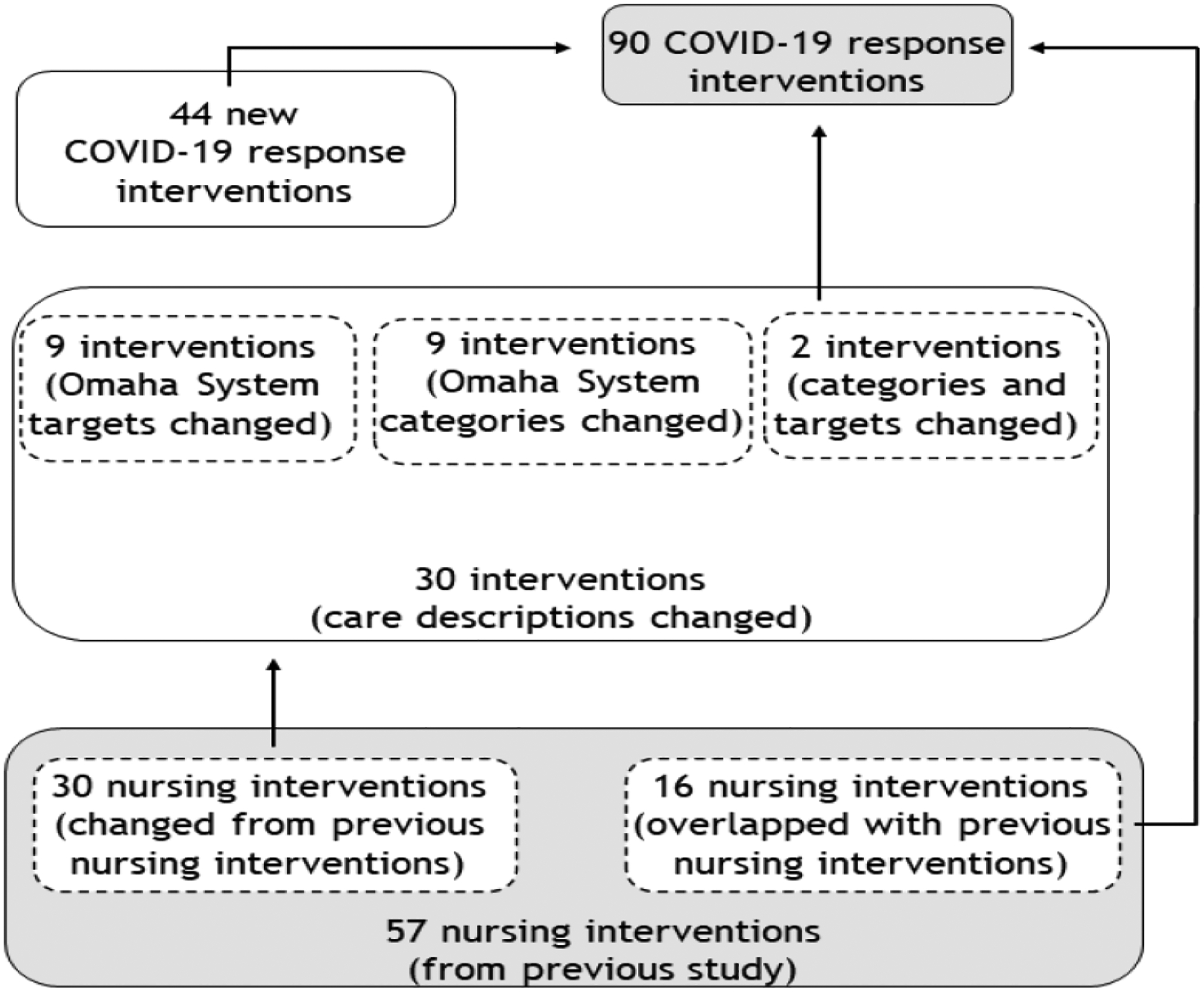
Comparison of COVID-19 Response Interventions with Nursing Home Interventions Prior to the Pandemic

A total of 90 nursing home COVID-19 response interventions that were specific to the Nursing Services roles were compared to the 57 nursing interventions from the previous study (Kang et al., in press, Appendix 1). Of the 90 COVID-19 response interventions, 16 overlapped with the nursing home interventions before the pandemic (Figure 2). The Omaha System categories and targets remained the same for these interventions, but care descriptions were adapted for the COVID-19 response.

Forty-four additional COVID-19 response interventions were identified (Figure 2 and Appendix 2), and this accounted for a 75% increase in the types of interventions over the 57 pre-pandemic interventions. Of these new COVID-19 response interventions, 16 (36%) involved *Teaching, Guidance and Counseling*, 16 (36%) involved *Treatments and Procedures*, 7 (16%) involved *Case Management*, and 5 (11%) involved *Surveillance*. The majority of the new interventions addressed the *infection precautions* (41%) and *sickness/injury care* (14%) targets. Care descriptions were changed for 30 COVID-19 response interventions. For 20 additional interventions, the Omaha System categories or targets were changed as well (Figure 2).

## DISCUSSION

Nursing home experts and informatics nurses collaborated on a COVID-19 Response Guideline that could be deployed in nursing home settings. The team used a crowdsourcing process to identify the six nursing home staff roles, and their extensive scope of interprofessional practice to respond to the COVID-19 pandemic. The comparison of the COVID-19 response interventions and the pre-pandemic interventions for RNs and LPN/LVNs, which was enabled by the Omaha System, showed the necessary adaptations and extensive collaboration among nurses needed in response to the pandemic. This COVID-19 Response Guideline is available in the public domain and may provide quick response guidance and references to nursing home staff. Furthermore, the guideline can be embedded within health information technology for routine care and documentation in nursing home settings, and documentation using the guideline will generate data to demonstrate and improve care quality and outcomes.

The COVID-19 Response Guideline included interprofessional interventions related to infection prevention and pandemic crisis management in nursing homes, and provided the most recent rationale regarding the application and management of personal protective equipment (PPE); COVID-19 symptom management; contact tracing and quarantine; and social distancing in congregate living settings. Such interventions aligned with crucial COVID-19 response interventions identified by multidisciplinary geriatric experts and updated COVID-19 regulations and guidance for nursing homes (Burn-Klug & Beaulieu, 2020; Levine et al., 2020; White et al., 2021). More importantly, the guideline included COVID-19 response interventions for all nursing home staff roles. Given the comprehensiveness of the guideline, it may serve as an efficient tool for educational purposes, either for internal protocol updates or for personal learning (Levine et al., 2020; White et al., 2021). Additionally, when the interventions are incorporated within health information technology, they can serve as care protocols and documentation templates as well as generating structured data for further analysis (Martin, 2005; Monsen, Swenson et al., 2017; Monsen, Vanderboom et al., 2017).

Extensive overlapping of interventions across roles (about 40% of interventions) indicated that it is critical to demonstrate partnership principles among the interprofessional roles and promote cohesive care delivery for effective COVID-19 management with limited resources (Bakerjian at al., 2021; Kolanowski et al., 2021; Levine et al., 2020; Scopetti et al., 2021; White et al., 2021). This culture change in nursing homes persisted well into the COVID-19 pandemic. Furthermore, the comparison identified additional nursing interventions related to the COVID-19 response, indicating the immense pressure that the pandemic has added to frontline nurses in nursing homes. Nurses were required to immediately triage and transfer residents, identify contacts of sick residents and staff, assess symptoms and signs of COVID-19, provide testing and critical care to residents, manage PPE and supplies, and teach PPE protocols and quarantine guidelines (Scopetti et al., 2021; White et al., 2021). To enable quality care under this pressure, individuals’ behavior and organizations’ cultures will need to change toward intra- and interprofessional partnership principles (Kennedy Oehlert, 2015). The COVID-19 Response Guideline is an exemplar of an informatics solution for building internal collaboration, as well as multidisciplinary partnerships with local health care systems, that are accessible to all nursing home staff using the Omaha System Guidelines web site or app (Omaha System Guidelines, 2021).

## CONCLUSION

The COVID-19 response in nursing homes depends on mutual support by dedicated professionals with overlapping responsibilities. The COVID-19 Response Guideline provides examples of how informatics can facilitate multidisciplinary partnership for nursing homes and other care settings. When partnership principles underlie guideline development and are incorporated within educational and documentation tools, there is potential to shift the existing hierarchical paradigm to one of mutual regard and meaningful progress toward shared goals. Future use of such guidelines for decision making and documentation related to infection prevention and control in nursing homes can improve nursing home care quality, and resident and population health.

## Supplementary Material

Appendices

## Figures and Tables

**Table 1:** Nursing Home Staff Roles

Nursing home staff roles	Employed professionals
Administration	Administrator, Nursing services director, Medical director
Nursing Services	Registered Nurse, Licensed Practical/Vocational Nurse, Certified Nursing Assistant
Therapeutic Services	Occupational Therapist/Assistant, Physical Therapist/Assistant, Speech-language Pathologist, Recreational Therapist/Assistant, Social Worker, Mental health professional/aide, Spiritual health professional/Chaplain
Dietary Services	Dietician, Dietary aide
Contract Services	Providers (Doctor of Medicine, Doctor of Osteopathic Medicine, Nurse Practitioner, Physician Assistant), Pharmacist, Podiatry, Dental, Lab, Radiology, Hospice
Environmental Services	

**Table 2:** Interventions within Scope for Nursing Home Staff Roles

Nursing home staff roles	Types of intervention within scope of practice (%)
Administration	100/117 (85%)
Nursing Services	90/117 (77%)
Therapeutic Services	70/117 (60%)
Dietary Services	52/117 (44%)
Contract Services	82/117 (70%)
Environmental Services	50/117 (43%)

Note. Reported data is based on the COVID-19 Response Guideline as of January 2022.
