# Supplementary material for: INTERPROFESSIONAL ROLES AND COLLABORATIONS TO ADDRESS COVID-19 PANDEMIC CHALLENGES IN NURSING HOMES
Source: Interdiscip J Partnersh Stud. Author manuscript; Available in PMC 2022 Oct 17. (PMC9574880; doi:10.24926/ijps.v9i1.4644)
Supplement: Appendices [file NIHMS1817750-supplement-Appendices.pdf]

APPENDICES

Appendix 1

Nursing Home Interventions Defined Prior to the Pandemic (N=57)

Problem	Category	Target	Care description
Bowel function	S	signs/symptoms of (s/sx) physical	Nurse assesses bowel function including feeding tube flushing and assessment
Circulation	S	s/sx physical	Nurse checks patient vital signs
Circulation	S	s/sx physical	Nurse manages IV site and function
Communicable/infectious condition	CM	infection precautions	Nurse manages infection precautions and isolation
Communicable/infectious condition	TP	infection precautions	Nurse performs hand hygiene
Consciousness	S	s/sx physical	Nurse assesses consciousness
Health care supervision	CM	communication	Nurse documents notes
Health care supervision	CM	communication	Nurse pages on-call/in-house primary providers
Health care supervision	CM	continuity of care	Nurse convenes or consults with interprofessional team including care conference
Health care supervision	CM	continuity of care	Nurse participates in report of patient condition and care among nursing department [e.g. shift report, report to other nurses or supervisor]
Health care supervision	CM	durable medical equipment	Nurse obtains or manages equipment [e.g. prepare device, check intravenous or feeding pump]

Health care supervision	CM	environment	Nurse manages non-patient-related work [e.g. new protocols/devices, technical issues]
Health care supervision	CM	medical/dental care	Nurse consults with in-house/on-call primary providers
Health care supervision	CM	medical/dental care	Nurse transcribes or manages orders
Health care supervision	CM	nursing care	Nurse reads written guidelines or evidence-based practice resources and policies
Health care supervision	CM	sickness/injury care	Nurse coordinates care [e.g. follow-up, appointments, Therapies, X-rays]
Health care supervision	CM	supplies	Nurse obtains or manages supplies [e.g. narcotics counts, set up nurse carts, obtain treatment materials, reorder supplies]
Health care supervision	S	communication	Nurse reviews patient chart and notes to gain comprehensive understanding of the patient health needs, care, and progress [e.g. review for pro re nata medications]
Health care supervision	S	laboratory findings	Nurse monitors patient lab results [e.g. blood glucose (BG) monitoring]
Health care supervision	S	s/sx physical	Nurse conducts thorough assessment of patient [e.g. admission assessment]
Health care supervision	TGC	anatomy/physiology	Nurse explains patient/family (p/f) about patient's condition in plain language
Health care supervision	TGC	end of life care	Nurse explains and guides p/f and certified nursing assistants (CNA) end of life care (including patient death)
Health care supervision	TGC	laboratory findings	Nurse explains p/f about laboratory tests and results [e.g. BG level check and management]
Health care supervision	TGC	nursing care	Nurse teaches student, new nurse, or CNA
Health care supervision	TGC	nursing care	Nurse explains p/f about treatment and procedure
Health care supervision	TGC	sickness/injury care	Nurse explains plan of care including discharge summary
Health care supervision	TGC	stimulation/nurturance	Nurse provides emotional support and comfort to p/f; also includes general conversation

Health care supervision	TP	nursing care	Nurse performs a treatment or procedure [e.g. suctioning]
Health care supervision	TP	specimen collection	Nurse obtains specimen
Medication regimen	CM	medication coordination/ordering	Nurse documents medications
Medication regimen	CM	medication set up	Nurse prepares medications
Medication regimen	S	medication action/side effects	Nurse asks patient about how medication is working, or observes patient for effects of medication
Medication regimen	TGC	medication action/side effects	Nurse explains p/f about medication action and side effects
Medication regimen	TP	medication administration	Nurse administers medications
Mental health	CM	behavior modification	Nurse manages p/f compliance and redirects behaviors
Mental health	TGC	coping skills	Nurse asks p/f about how patient copes with illness and hospitalization
Mental health	TP	bonding/attachment	Nurse holds patient's hand or provides calming touch
Neighborhood/workplace safety	CM	safety	Nurse manages conditions that promote patient safety [e.g. fall managements, fall interventions]
Neighborhood/workplace safety	S	environment	Nurse monitors and maintains patient’s room and hall
Neighborhood/workplace safety	TP	environment	Nurse maintains cleanliness and order in room or hall
Neuro-musculo-skeletal function	S	s/sx physical	Nurse assesses neuro-musculo-skeletal function [e.g. range of motion]
Neuro-musculo-skeletal function	TP	positioning	Nurse repositions patient
Nutrition	CM	dietary management	Nurse manages dietary needs [e.g. manages meal tickets, confirms patient diet]
Nutrition	TGC	dietary management	Nurse teaches p/f about diet

Nutrition	TP	feeding procedures	Nurse gives food or helps patient to eat [e.g. nutritional supplement, total parenteral nutrition, or tube feeding]
Pain	S	s/sx physical	Nurse observes or asks patient about pain
Personal care	TP	bladder care	Nurse manages urinary catheter (insertion to discontinuation)
Personal care	TP	ostomy care	Nurse assists with special bowel care [e.g. ostomy]
Personal care	TP	personal hygiene	Nurse gives personal hygiene care including bath, toileting, or massage
Physical activity	TP	mobility/transfer	Nurse manages physical patient transfer
Respiration	TP	respiratory care	Nurse helps patient with deep breathing, coughing or respirator
Skin	S	s/sx physical	Nurse assesses skin or wound
Skin	TP	dressing change/wound care	Nurse provides wound care
Sleep and rest patterns	TP	rest/sleep	Nurse helps patient become comfortable for sleep [e.g. rest, daytime nap, or bedtime]
Spirituality	CM	spiritual care	Nurse refers the patient to chaplaincy for spiritual care
Spirituality	TGC	spiritual care	Nurse provides holistic care including spiritual support
Urinary function	S	s/sx physical	Nurse assesses urinary function [e.g. bladder scan]

Note. Abbreviations: Case Management (CM), Teaching, Guidance, and Counseling (TGC), Treatments and Procedures (TP), Surveillance (S)

Appendix 2

The New Nursing Home COVID-19 Response Interventions (N=44) in Scope of Practice by Nursing Home Staff Role (Problem: Communicable/infectious condition)

Category	Target	Care description	Administration	Nursing Services	Therapeutic Services	Dietary Services	Contract Services	Environmental Services
CM	medical/dental care	What to do if you are sick, when to seek medical care	Y	Y	Y	Y	Y	Y
CM	sickness/injury care	Acute care visitors' policy exception to accompany person with ID	Y	Y	Y	Y	Y	Y
TGC	continuity of care	Identify and protect vulnerable people	Y	Y	Y	Y	Y	Y
TGC	infection precautions	Disseminate messages to the public, address misinformation	Y	Y	Y	Y	Y	Y
TGC	infection precautions	Modify, postpone, or cancel large events	Y	Y	Y	Y	Y	Y
TGC	infection precautions	Personal readiness	Y	Y	Y	Y	Y	Y
TGC	infection precautions	Adhere to physical distancing recommendations	Y	Y	Y	Y	Y	Y
TGC	infection precautions	Quarantine guidelines to prevent spread	Y	Y	Y	Y	Y	Y
TGC	infection precautions	Universal facemasks	Y	Y	Y	Y	Y	Y

TGC	interaction	Counter stigma and discrimination	Y	Y	Y	Y	Y	Y
TGC	sickness/injury care	Prepare for surge in patients	Y	Y	Y	Y	Y	Y
TGC	stress management	Promote healthy functioning activities	Y	Y	Y	Y	Y	Y
TGC	support system	Build formal and informal neighborhood/community support systems	Y	Y	Y	Y	Y	Y
TP	behavior modification	Cover cough, avoid touching face	Y	Y	Y	Y	Y	Y
TP	infection precautions	Adhere to physical distancing recommendations	Y	Y	Y	Y	Y	Y
TP	infection precautions	Stay home when sick	Y	Y	Y	Y	Y	Y
TP	sickness/injury care	Personal response of ill community members	Y	Y	Y	Y	Y	Y
TP	sickness/injury care	Prepare for surge in patients	Y	Y	Y	Y	Y	Y
TP	wellness	Interpersonal support; hydration, nourishment, exercise, sleep; limit media, limit substance use	Y	Y	Y	Y	Y	Y
S	infection precautions	Identify contacts of sick people	Y	Y	Y	Y	Y	Y
S	infection precautions	Adhere to physical distancing recommendations	Y	Y	Y	Y	Y	Y
CM	infection precautions	Encourage virtual transactions e.g. telehealth, teleeducation,	Y	Y	Y	N	Y	N

		telesupport, telebusiness, phone, mail, video						
CM	medical/dental care	Standardized care protocols	Y	Y	Y	N	Y	N
CM	other community resources	Clothing, face masks, internet, telephone	Y	Y	Y	N	Y	N
CM	medication coordination/ordering	Vaccines	Y	Y	N	N	N	N
CM	signs/symptoms-physical	Triage, place, and discharge patients	Y	Y	N	N	N	N
TGC	infection precautions	Transmission routes and sources	Y	Y	Y	N	Y	N
TGC	infection precautions	Protocols for cleaning, reusing, and extending the life of medical supplies and equipment	Y	Y	Y	N	Y	Y
TGC	medication action/side effects	Vaccines	Y	Y	N	N	Y	N
TGC	interaction	Health equity	N	Y	Y	N	N	N
TGC	sickness/injury care	Give sick household members their own room and have only one member care for them	Y	Y	N	N	N	N
TP	infection precautions	Protocols for cleaning, reusing, and extending the life of medical supplies and equipment	Y	Y	Y	N	N	Y

TP	infection precautions	Avoid aerosol generating procedures	Y	Y	Y	N	Y	N
TP	end of life care	Symptom relief/palliative care	N	Y	Y	N	Y	N
TP	end of life care	Provide palliative care	N	Y	Y	N	Y	N
TP	infection precautions	Take precautions when performing aerosol-generating procedures	N	Y	Y	N	Y	N
TP	infection precautions	Placement of persons receiving treatment	Y	Y	N	N	N	N
TP	interaction	Health equity	N	Y	Y	N	N	N
TP	medical/dental care	Fluid management	N	Y	N	N	Y	N
TP	sickness/injury care	Give sick household members their own room and have only one member care for them	Y	Y	N	N	N	N
TP	medication administration	Vaccines	N	Y	N	N	N	N
S	infection precautions	Test for disease	Y	Y	N	N	Y	N
S	wellness	Immunization status	Y	Y	N	N	N	N
S	signs/symptoms -physical	Fever, cough, and shortness of breath	Y	Y	Y	Y	Y	Y

Note. Abbreviations: Case Management (CM), Teaching, Guidance, and Counseling (TGC), Treatments and Procedures (TP), Surveillance (S)
